# The economic burden of dementia in China, 1990–2030: implications for health policy

**DOI:** 10.2471/BLT.15.167726

**Published:** 2016-10-18

**Authors:** Junfang Xu, Jian Wang, Anders Wimo, Laura Fratiglioni, Chengxuan Qiu

**Affiliations:** a Research Centre for Public Health, Tsinghua University, Beijing, China.; b Centre for Health Economic Experiments and Public Policy, Shandong University, Jinan, China.; c Division of Neurogeriatrics, Karolinska Institutet, Stockholm, Sweden.; d Ageing Research Centre, Department of Neurobiology, Care Sciences and Society, Karolinska Institutet, Stockholm University, Gävlegatan 16, 113 30 Stockholm, Sweden.

## Abstract

**Objective:**

To quantify and predict the economic burden of dementia in China for the periods 1990–2010 and 2020–2030, respectively, and discuss the potential implications for national public health policy.

**Methods:**

Using a societal, prevalence-based, gross cost-of-illness approach and data from multiple sources, we estimated or predicted total annual economic costs of dementia in China. We included direct medical costs in outpatient and inpatient settings, direct non-medical costs – e.g. the costs of transportation – and indirect costs due to loss of productivity. We excluded comorbidity-related costs.

**Findings:**

The estimated total annual costs of dementia in China increased from 0.9 billion United States dollars (US$) in 1990 to US$ 47.2 billion in 2010 and were predicted to reach US$ 69.0 billion in 2020 and US$ 114.2 billion in 2030. The costs of informal care accounted for 94.4%, 92.9% and 81.3% of the total estimated costs in 1990, 2000 and 2010, respectively. In China, population ageing and the increasing prevalence of dementia were the main drivers for the increasing predicted costs of dementia between 2010 and 2020, and population ageing was the major factor contributing to the growth of dementia costs between 2020 and 2030.

**Conclusion:**

In China, demographic and epidemiological transitions have driven the growth observed in the economic costs of dementia since the 1990s. If the future costs of dementia are to be reduced, China needs a nationwide dementia action plan to develop an integrated health and social care system and to promote primary and secondary prevention.

## Résumé

**Objectif:**

Calculer et prévoir la charge économique de la démence en Chine pour les périodes s'étendant de 1990 à 2010 et de 2020 à 2030 respectivement, et analyser les conséquences potentielles sur la politique nationale de santé publique.

**Méthodes:**

À l'aide d'une approche sociétale relative au coût brut de la maladie fondée sur la prévalence et de données provenant de différentes sources, nous avons effectué une estimation et une prévision du coût économique annuel total de la démence en Chine. Nous avons pris en compte les coûts médicaux directs dans les services de consultation externe et en milieu hospitalier, les coûts non médicaux directs – les coûts de transport, par exemple – et les coûts indirects dus à une perte de productivité. Nous n'avons pas tenu compte des coûts liés à la comorbidité.

**Résultats:**

D'après nos estimations, le coût annuel total de la démence en Chine est passé de 0,9 milliard de dollars des États-Unis (US$) en 1990 à 47,2 milliards d'US$ en 2010, et nos prévisions indiquent qu'il atteindra 69,0 milliards d'US$ en 2020 et 114,2 milliards d'US$ en 2030. Le coût des soins informels représente 94,4%, 92,9% et 81,3% du coût total estimé pour les années 1990, 2000 et 2010, respectivement. En Chine, le vieillissement de la population et la prévalence croissante de la démence étaient les principaux facteurs de l'augmentation des coûts liés à la démence prévus entre 2010 et 2020, et le vieillissement de la population était le principal facteur contribuant à l'augmentation des coûts liés à la démence entre 2020 et 2030.

**Conclusion:**

En Chine, les transitions démographiques et épidémiologiques ont entraîné depuis les années 90 une augmentation des coûts économiques liés à la démence. Afin de réduire les futurs coûts liés à la démence, la Chine doit élaborer un plan d'action d'ampleur nationale à l'égard de la démence pour pouvoir mettre en place un système de santé et de services sociaux intégré et promouvoir la prévention primaire et secondaire.

## Resumen

**Objetivo:**

Cuantificar y predecir la carga económica de la demencia en China durante los periodos comprendidos entre 1990 y 2010 y entre 2020 y 2030, respectivamente, y analizar las posibles implicaciones para la política sanitaria pública del país.

**Métodos:**

Utilizando un enfoque social, basado en la prevalencia y coste bruto de la enfermedad, así como información de varias fuentes, se estimó o se predijo el total de costes económicos anuales de la demencia en China. Se incluyeron los costes médicos directos en entornos ambulatorios y hospitalarios, los costes no médicos directos (por ejemplo, los costes de transporte) y los costes indirectos derivados de la pérdida de productividad. No se incluyeron los costes relacionados con la comorbilidad.

**Resultados:**

El total de costes anuales estimados de la demencia en China aumentó de 900 millones de dólares estadounidenses (USD) en 1990 a 47 200 millones de USD en 2010, y se prevé que aumentarán hasta los 69 000 millones de USD en 2020 y los 114 200 millones de USD en 2030. Los costes de la atención informal sumaban un 94,4%, un 92,9% y un 81,3% del total de costes estimados en 1990, 2000 y 2010, respectivamente. En China, el envejecimiento demográfico y la creciente prevalencia de la demencia fueron los principales impulsores del aumento de los costes de la demencia previstos entre 2010 y 2020, y el envejecimiento demográfico fue el principal factor que contribuyó al crecimiento de los costes de la demencia entre 2020 y 2030.

**Conclusión:**

En China, las transiciones demográficas y epidemiológicas han impulsado el crecimiento observado en los costes económicos de la demencia desde la década de los 90. Si el objetivo es reducir los futuros costes de la demencia, China necesita un plan de acción nacional contra la demencia para desarrollar un sistema de atención sanitaria y social integrado y fomentar la prevención primaria y secundaria.

## ملخص

**الهدف:**

وضع تقدير كَميّ وتنبؤ بحجم العبء الاقتصادي للخَرَف في الصين للفترات ما بين 1990-2010 و2020-2030، على التوالي، ومناقشة الآثار المحتملة للسياسة الصحية الوطنية العامة.

**الطريقة:**

باستخدام نهج مجتمعي قائم على الانتشار، ومعرفة إجمالي تكلفة الأمراض بالإضافة إلى البيانات المأخوذة من مصادر متعددة، فقد تمكنا من وضع تقدير أو تنبؤ بإجمالي التكاليف الاقتصادية السنوية للخَرَف في الصين. وقما بإدراج التكاليف الطبية المباشرة في العيادات الخارجية والداخلية، بالإضافة إلى التكاليف غير الطبية المباشرة – مثل تكاليف النقل – والتكاليف غير المباشرة الناتجة عن فقدان الإنتاجية. وقمنا باستثناء التكاليف المتعلقة بالمراضة المشتركة.

**النتائج:**

ارتفعت التكاليف السنوية الإجمالية المقدرة للخَرَف في الصين من 0.9 مليار دولار أمريكي في عام 1990 إلى 47.2 مليار دولار أمريكي في عام 2010 و من المتوقع أن تصل إلى 69.0 مليار في عام 2020 و114.2 مليار دولار أمريكي في عام 2030. وشكلّت تكاليف الرعاية غير الرسمية ما يساوي 94.4‏% و‏92.9% و‏81.3% من إجمالي التكاليف المقدرة في أعوام 1990 و2000 و2010 على الترتيب. لقد كانت شيخوخة السكان وتزايد انتشار الخَرَف في الصين هما العاملان الرئيسيان لزيادة التكاليف المتوقعة للخَرَف، وذلك في الفترة ما بين عامي 2010 و2020 ، فيما كانت شيخوخة السكان هي العامل الرئيسي المساهم في زيادة تكاليف الخَرَف في الفترة ما بين عامي 2020 و2030.

**الاستنتاج:**

أدت التحولات الديمغرافية والوبائية في الصين إلى حدوث نمو ملحوظ في التكاليف الاقتصادية للخَرَف منذ تسعينيات القرن العشرين. وإذا كانت هناك اتجاه لخفض تكاليف الخَرَف في المستقبل، فإن الصين بحاجة إلى وضع خطة عمل للتعامل مع الخَرَف على مستوى البلاد بهدف تطوير نظام متكامل للرعاية الصحية والاجتماعية، فضلاً عن تعزيز الوقاية الأولية والثانوية.

## 摘要

**目的:**

旨在量化和预测中国境内 1990 年至 2010 年和 2020 年至 2030 年期间分别因痴呆症带来的经济负担，并且讨论对国家公共卫生政策的潜在影响。

**方法:**

我们使用一种基于患病率的社会方法——总疾病成本推算法以及来自多个来源的数据，估计或预测了中国境内痴呆症的年度经济总成本。 我们包括了门诊和住院环境中的直接医疗成本、直接非医疗成本（例如交通成本）以及由于生产力损失产生的间接成本。 我们未包括与合并症相关的成本。

**结果:**

据估计，中国境内痴呆症的年度总成本从 1990 年的 9 亿美元增加到 2010 年的 472 亿美元，预计到 2020 年将达到 690 亿美元，2030 年将达到 1142 亿美元。非正式护理的成本在 1990 年、2000 年和 2010 年分别占估计总成本的 94.4％、92.9％ 和 81.3％。 在中国，人口老龄化和痴呆症患病率的增加是 2010 年至 2020 年间痴呆症预期成本增加的主要原因，人口老龄化是导致 2020 年至 2030 年间痴呆症成本增长的主要因素。

**结论:**

在中国，自二十世纪九十年代以来，人口和流行病学方面的转变推动了痴呆症经济成本的增长。 如果要降低未来的痴呆成本，中国需要通过全国性的痴呆症行动计划来发展综合型卫生与社会关怀体系并且促进一级和二级预防。

## Резюме

**Цель:**

Дать количественную оценку экономического бремени деменции в Китае за период с 1990 по 2010 год и спрогнозировать его на период с 2020 по 2030 год, а также изучить возможные последствия для национальной политики в области общественного здравоохранения.

**Методы:**

Используя подход, основанный на подсчете общей стоимости болезни для общества в зависимости от распространенности, и данные из нескольких источников, авторы определили или предсказали суммарные годовые экономические издержки, вызванные деменцией, в Китае. Учитывались прямые медицинские расходы на лечение в условиях амбулатории и стационара, прямые немедицинские расходы (например, транспортные расходы) и косвенные издержки, связанные со снижением производительности труда. Авторы не учитывали расходы, вызванные сопутствующими заболеваниями.

**Результаты:**

Было подсчитано, что суммарная годовая стоимость деменции в Китае увеличилась с 0,9 млрд долларов США в 1990 году до 47,2 млрд долларов США в 2010 году; согласно прогнозам стоимость достигнет 69 млрд долларов в 2020 году и 114,2 млрд долларов в 2030 году. Издержки неофициального ухода составили 94,4; 92,9 и 81,3% от общей подсчитанной суммы расходов в 1990, 2000 и 2010 годах соответственно. В Китае основными причинами увеличения прогнозируемой стоимости деменции в период с 2010 по 2020 год стали старение населения и рост распространенности деменции; авторы назвали старение населения главным фактором, который обусловит увеличение стоимости деменции между 2020 и 2030 годами.

**Вывод:**

В Китае демографический и эпидемиологический переходы стали причиной увеличения экономических издержек, вызванных деменцией, с 1990-х годов. Чтобы снизить будущие расходы, Китаю требуется общенациональный план действий по борьбе с деменцией для развития комплексной системы здравоохранения и социальной помощи и для содействия распространению первичной и вторичной профилактики.

## Introduction

According to the 2013 Alzheimer’s Disease International report, about 44.4 million people were living with dementia in 2013 and this number is expected to reach an estimated 75.6 million by 2030.^1^ In China, which has the largest population of people with dementia, the prevalence of dementia appears to have increased steadily between 1990 and 2010.^2^^,^^3^ However, this trend might be partly attributed to temporal variations in the methods used to estimate such prevalence.^4^ The results of a national survey in 2008–2009 indicated that dementia was more common in rural areas than in urban settings.^5^ Given the rapid growth of the elderly population in China,^6^ dementia is expected to pose tremendous challenges to the national health-care system and to the sustainable development of the national economy.

Most cost-of-illness studies for dementia have been carried out in high-income countries such as Sweden, the United Kingdom of Great Britain and Northern Ireland and the United States of America.^7^^–^^11^ The economic costs of dementia in China – which have yet to be investigated in detail – are likely to differ, both in magnitude and type, from those in such distant high-income countries.

In this study, we sought to estimate and predict the costs of dementia in China for the periods 1990–2010 and 2020–2030, respectively. It was hoped that, by quantifying the economic costs of dementia, Chinese policy-makers would be motivated to develop a nationwide action plan, prioritize policies on dementia-related care and research and reduce the economic and societal burdens of dementia in China.

## Methods

In this cost-of-illness study, we used a prevalence-based, bottom-up approach to quantify or predict the costs of dementia in China between 1990 and 2030, from a societal perspective. We categorized all the costs into three classes:^12^^,^^13^ (i) direct medical costs, that is goods and service costs related to the diagnosis and treatment of inpatients and outpatients with dementia; (ii) direct non-medical costs, that is transport costs and costs related to formal care in nursing homes or informal care at home; and (iii) indirect costs resulting from dementia-attributable loss of productivity.

### Data sources

We used multiple data sources for all estimates. We used age-specific prevalence of dementia in China, for the period 1990–2010, derived from a comprehensive systematic review.^3^ From the electronic health records of the facilities, we collected cost data for patients with diagnosed dementia who were admitted either to the Shandong Centre for Mental Health – the only provincial psychiatric hospital in the eastern province of Shandong – between 1 January 2005 and 31 March 2014 or to the Daizhuang Psychiatric Hospital – one of the oldest psychiatric hospitals in China and also in Shandong province – between 1 January 2012 and 30 September 2014. The routine electronic health records include sociodemographic data and data on clinical diagnosis and disease classification, itemized costs, e.g. for drugs, examinations and beds. In each of the two study facilities, dementia was diagnosed and defined according to the *International statistical classification of diseases and related health problems, 10th revision*.^14^ We excluded 26 patients with dementia who were diagnosed as having other chronic conditions that needed treatment, e.g. anxiety, diabetes or hypertension, leaving data from the records of 146 patients with dementia in our analysis. We also searched the China National Knowledge Infrastructure, PubMed and Wanfang bibliographic databases for studies, on the use of health resource by people with dementia in China, published between 1 January 1990 and 31 July 2015. The search terms included “Alzheimer’s disease”, “China”, “cost burden”, “dementia”, “economic burden”, “formal care” and “informal care”. We obtained costs for outpatient visits and transportation from a published study.^15^ Data on demographics and wages came from the *China Statistical Yearbook 2015*.^16^ The United Nations population projections for China^17^ and predictions of the prevalence of dementia based on data from a systematic review^3^ were used to estimate the total numbers of people in China who would have dementia in 2020 and 2030.

### Cost estimates

For our estimates we included costs for hospitalization, formal care, informal care, loss of productivity, outpatient care and transportation. Our estimates of hospitalization costs, which included the costs of all medicines, clinical examinations, specialist consultations and bed care, were based on the mean values of the costs recorded in the electronic health records of the two study facilities and the values given in a published article^15^ – all weighted according to the sample sizes. Our estimates of the costs of formal care were similarly weighted mean values based on published data.^18^^,^^19^ Informal care costs were estimated assuming that a carer, who might otherwise be earning the national mean salary,^13^ spent a mean of 6.3 hours per day^20^ giving care to each dementia case in informal care at home. We assumed that, during the observational periods, 86% of Chinese dementia cases were receiving informal care at home and that 4.9% of such cases would seek formal care.^13^^,^^21^ We estimated the numbers of disability-adjusted life-years (DALYs) lost because of dementia using each case’s dementia severity score, assessed using the Global Deterioration Scale,^22^ and different weights for each of seven levels of severity.^23^ Costs of the productivity lost because of dementia-related disability were then estimated. For these estimations, we assumed that each person aged at least 60 years had a mean productivity weight of 0.1 and we used an annual discount rate of 3.5% to adjust the costs to 2015 values.^24^ Outpatient care costs included the costs of treatments and specialist consultations received in clinics or at home.^15^ Transportation costs comprised the costs travelling to and from medical centres.^15^

All the estimated costs were converted to United States dollar (US$) values in January 2015, when US$ 1 was equivalent to about 6.2 Chinese yuan.

### Statistical analysis

We estimated the numbers of people with dementia in China in 1990, 2000 and 2010 by multiplying the age-specific prevalence of dementia^3^ by the corresponding numbers of people in each age group in the population. For our predictions for 2020 and 2030, we used age-specific prevalence derived using a regression model and the relevant data from a comprehensive review.^3^ We estimated the total annual costs of dementia by multiplying the mean costs per patient by the total number of patients with dementia. Total annual costs for 2020 and 2030 were projected using a dynamic general disequilibrium model^25^ and assuming that the use of health resources by a dementia case was constant while the elderly population grew and the age-specific prevalence of dementia varied over time. We employed the Laspeyres decomposition method to estimate the relative contributions made by the ageing population and changes in the age-specific prevalence of dementia to the predicted future costs of dementia.^26^ When the relevant data on medical costs for particular years were missing, we assumed that those costs would have increased by the same amount as the per-capita gross domestic product (GDP).

We conducted multiple sensitivity analyses to assess the impact of variations in the key input parameters on our primary estimates. Specifically, we estimated the total costs of dementia by (i) using prevalence data derived from a different systematic review^2^ that yielded more conservative estimates of the prevalence of dementia than those that we used^3^ for our primary estimates; (ii) assuming that medical costs would increase 5% every year; (iii) using the minimum and maximum values – instead of the overall mean – for the hours spent on informal care;^27^ (iv) using the means of the minimum and maximum values recorded, in China’s 22 provinces, five autonomous regions and four municipalities, for an informal carer’s wages – instead of the overall national mean value; (v) assuming that dementia cases aged at least 60 years had a mean productivity weight of 0.50^27^ instead of 0.1; (vi) assuming that 70% or 99% of people with dementia – instead of 86% – would live at home;^7^ and (vii) assuming that 60.4% or 0.4% of dementia patients – instead of 4.9% – would seek professional care.^21^

## Results

The total number of people with dementia in China was estimated to be about 3.5 million in 1990, 5.1 million in 2000 and 9.6 million in 2010 (Table 1). The overall prevalence of dementia among people aged at least 60 years was projected to increase from 5.8% in 2020 to 6.7% in 2030 (Table 2). The total number of people with dementia in China was projected to reach 14.1 million by 2020 and 23.3 million by 2030.

**Table 1:** Estimated numbers of people with dementia, China, 1990, 2000 and 2010

Variable	Thousands of people with dementia^a^		
	1990 *n* = 2479.7	2000 *n* = 5148.4	2010* n* = 9615.6
**Age in years**			
60–64	314.3	454.6	792.0
65–69	462.0	726.9	1060.7
70–74	654.7	989.7	1569.5
75–79	726.1	1099.1	2027.4
80–84	655.0	948.3	1956.5
85–89	435.7	598.3	1369.7
≥ 90	231.9	331.6	839.7
**Sex**			
Male	1313.1	1942.8	3628.5
Female	2166.6	3205.6	5987.1
**Residence**			
Urban	2109.8	3121.5	5829.9
Rural	1370.0	2026.9	3785.7

NOTE: Inconsistencies arise in some values due to rounding.

^a^ Estimates based on a systematic review of the prevalence of dementia in China.^3^

**Table 2:** Predicted age-specific prevalence of dementia and numbers of people with dementia, China, 2020 and 2030

Age in years	2020			2030	
	Prevalence (%)	Thousands of cases		Prevalence (%)	Thousands of cases
60–64	1.5	1 121.1		1.7	1 879.0
65–69	3.0	2 117.3		3.4	2 966.8
70–74	5.3	2 340.7		6.0	3 661.7
75–79	9.7	2 593.2		11.0	5 477.5
80–84	16.6	2 717.8		18.8	4 547.9
85–89	27.8	2 096.8		31.5	2 997.0
≥ 90	47.4	1 082.1		53.9	1 760.8
≥ 60^a^	5.8	14 069.0		6.7	23 290.7

^a^ The values shown cover all of the expected dementia cases aged at least 60 years.

Between 1990 and 2010, the mean annual costs of formal and informal care for each dementia case increased more than 60-fold and more than 18-fold, respectively (Table 3). Over the same period, the national total annual costs of dementia increased more than 50-fold, from about US$ 0.9 billion in 1990 to approximately US$ 47.2 billion, or about 0.7% of China’s GDP in 2010.

**Table 3:** Estimated costs of dementia, China, 1990, 2000 and 2010

Cost item	Year		
	1990	2000	2010
**Costs per case (US$)**			
Direct medical costs			
Hospitalization cost	37.1	370.0	1 004.0
Outpatient care cost	0.5	5.0	148.8
Direct non-medical costs			
Nursing home care	47.4	222.4	4 468.1
Transportation	0.5	4.9	143.8
Informal care	271.5	1184.0	4 635.4
Indirect costs			
Cost due to DALYs lost	4.8	22.9	87.6
Total for cases living at home	314.4	1 586.8	6 019.6
Total for cases living in nursing homes	90.3	625.2	5 852.3
**National costs (US$ millions)**			
Direct medical costs			
Hospitalization cost	6.3	93.3	473.0
Outpatient care cost	1.8	26.1	1 431.0
Direct non-medical costs			
Nursing home care	23.1	160.3	6 014.9
Transportation	0.1	1.2	67.8
Informal care	812.4	5 242.3	38 332.0
Indirect costs			
Cost due to DALYs lost	16.7	118.1	842.3
Total for all cases	860.4	5 641.4	47 161.0
Sex of case			
Men	324.7	2 128.6	17 796.5
Women	535.7	3 512.2	29 364.2
Residence of case			
Urban	514.6	3 375.0	28 578.9
Rural	345.8	2 266.4	18 582.1

DALYs: disability-adjusted life-years; US$: United States dollars.

Note: All the estimated costs were converted to United States dollar (US$) values in January 2015, when US$ 1 was equivalent to about 6.2 Chinese yuan.

**Fig. 1:**
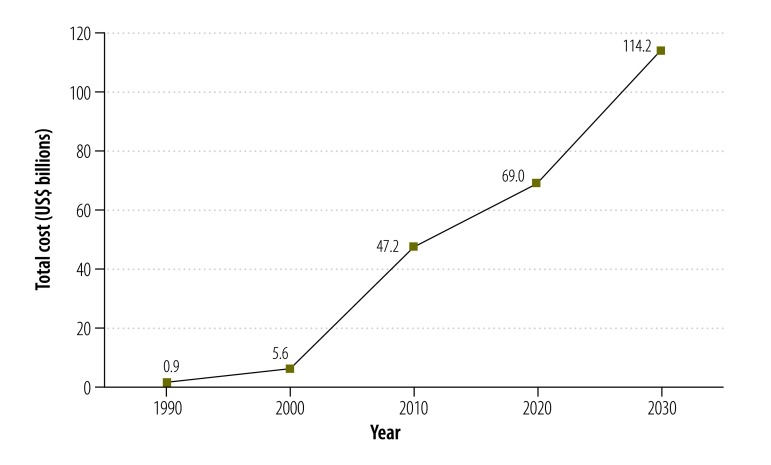
Estimated total annual costs of dementia, China, 1990–2030

**Fig. 2:**
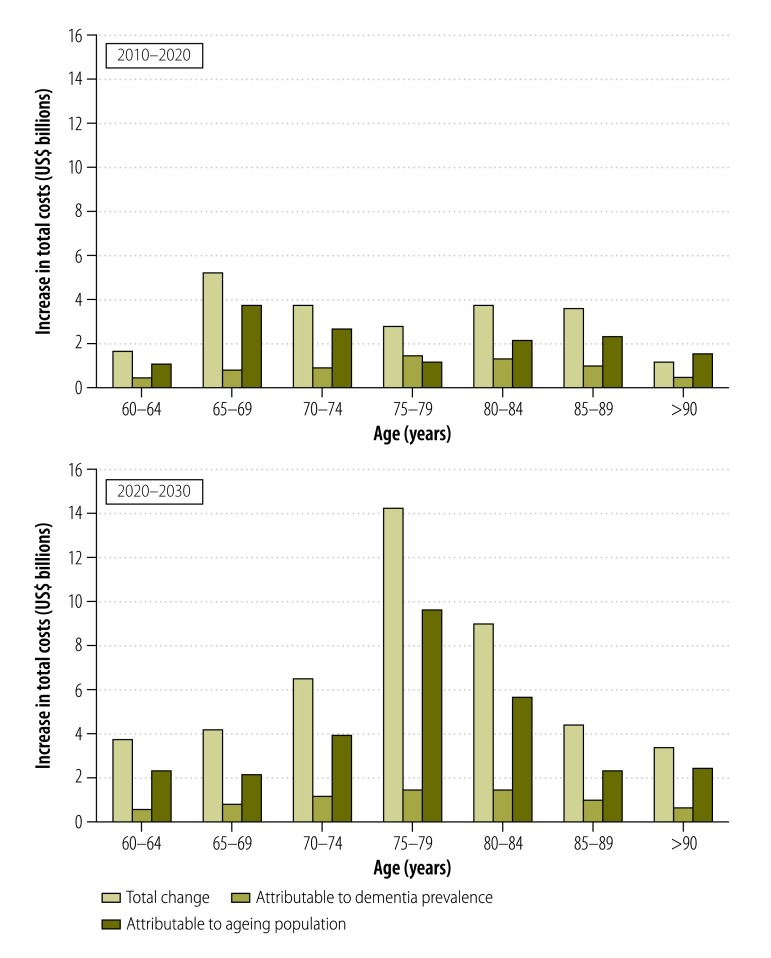
Attribution of the causes of the changes in total annual costs of dementia care, China, 2010–2030

The total costs of dementia were projected to reach US$ 69.0 billion in 2020 and US$ 114.2 billion in 2030 (Fig. 1). The ageing population and increases in dementia prevalence appeared to be the major driving factors for the high costs of dementia in 2010–2020 and the ageing population also appeared to be the dominant force behind the growth of dementia costs between 2020 and 2030 (Fig. 2).

US$: United States dollars.

Note: All the estimated costs were converted to United States dollar (US$) values in January 2015 – when US$ 1 was equivalent to about 6.2 Chinese yuan.

US$: United States dollars.

Note: All the estimated costs were converted to United States dollar (US$) values in January 2015 – when US$ 1 was equivalent to about 6.2 Chinese yuan.

**Fig. 3:**
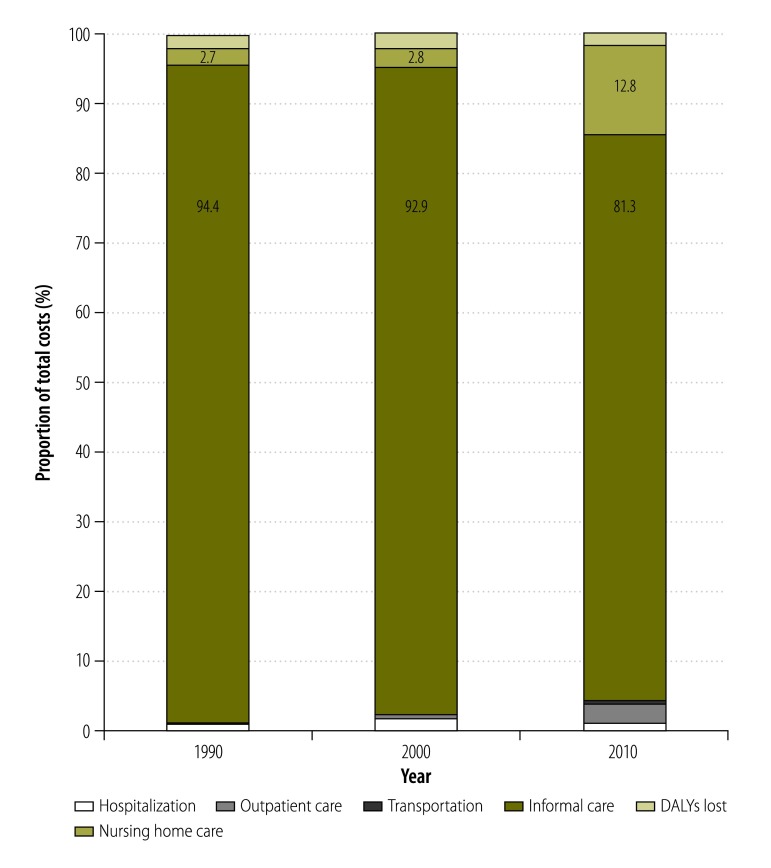
Relative contributions made by six cost items to the total annual costs of dementia care, China, 1990, 2000 and 2010

According to our estimates, the costs of informal care for dementia accounted for 94.4%, the total costs of dementia care in China in 1990, decreasing to 92.9% in 2000 and 81.3% in 2010 (Fig. 3). In contrast, the costs of formal care accounted for just 2.7% of the total costs in 1990, increasing to 2.8% in 2000 and 12.8% in 2010. Hospitalization costs accounted for only about 1% of total costs in each year between 1990 and 2010.

DALYs: disability-adjusted life-years.

The results of our sensitivity analyses indicated that variations in informal care hours, prevalence of dementia and productivity weighting had a substantial impact on estimates of the total costs of dementia, whereas changes in medical prices, wages and the proportions of patients living at home or seeking professional care had relatively little impact on such estimates (Table 4).

**Table 4:** Changes in the estimated annual costs of dementia according to variations in the key parameters considered in sensitivity analyses, China, 1990, 2000, 2010, 2020 and 2030

Parameter varied	Changes in estimated total costs,^a^ US$ billions (%)				
	1990	2000	2010	2020	2030
**Prevalence of dementia^b^**	−0.5 (−62.3)	−1.6 (−27.8)	−15.8 (−33.5)	−25.0 (−27.6)	−34.6 (−23.1)
**Medical costs**					
5% annual increase between 2010 and 2030, instead of 0%	ND	ND	ND	+7.3 (+9.6)	+31.7 (+21.7)
**Informal care in hours per day**					
2.5 instead of 6.3	−0.5 (−56.9)	−3.2 (−56.0)	−23.1 (−49.0)	−33.8 (−49.0)	−56.0 (−49.0)
8.9 instead of 6.3	+0.3 (+39.0)	+2.2 (+38.4)	+15.8 (+33.5)	+23.1 (+33.5)	+38.3 (+33.5)
**Wage for caregivers^c^**					
Minimum instead of mean	ND	−1.2 (−21.4)	−9.3 (−19.8)	−13.7 (−19.8)	−22.6 (−19.8)
Maximum instead of mean	ND	+2.1 (+37.6)	+5.5 (+11.7)	+8.1 (+11.7)	+13.4 (+11.7)
**Productivity weight for those aged ≥ 60 years**					
0.5 instead of 0.1	+0.2 (+27.2)	+1.5 (+27.5)	+11.2 (+23.8)	+16.4 (+23.8)	+27.2 (+23.8)
**Percentage of cases living at home**					
70% instead of 86%	−0.1 (−14.5)	−0.8 (−14.0)	−0.3 (−0.5)	−0.4 (−0.5)	−0.6 (−0.5)
99% instead of 86%	+0.1 (+11.8)	+0.6 (+11.4)	+0.2 (+0.4)	+0.3 (+0.4)	+0.5 (+0.4)
**Percentage of cases seeking professional treatment**					
60.4% instead of 4.9%	+0.1 (+8.5)	+1.1 (+19.0)	+6.1 (+13.0)	+9.0 (+13.0)	+14.8 (+13.0)
0.4% instead of 4.9%	+0.01 (+0.6)	+0.1 (+1.5)	+0.5 (+1.1)	+0.7 (+1.1)	+1.2 (+1.1)

ND: not determined; US$: United States dollars.

^a^ Compared with the primary estimates of costs.

^b^ Prevalence values given in one systematic review,^2^ that is, 1.3% in 1990, 2.9% in 2000 and 3.6% in 2010, instead of the values used for the primary estimates, that is, 4.9% in 1990, 5.4% in 2000 and 7.5% in 2010.^3^

^c^ We used the national mean of the minimum per-capita wages recorded in China’s 22 provinces, five autonomous regions and four municipalities, that is, US$ 1006.6 in 2000 and US$ 3872.6 in 2010, or the national mean of the corresponding maximum wages, that is, US$ 1812.6 in 2000 and US$ 5774.4 in 2010, instead of the overall national mean wages of US$ 1505.3 in 2000 and US$ 5893.4 in 2010. We predicted the corresponding values for 2020 and 2030 from the values for 2010.

Note: All the estimated costs were converted to United States dollar (US$) values in January 2015, when US$ 1 was equivalent to about 6.2 Chinese yuan

## Discussion

### Increasing costs of dementia

In China, from an age of 60 years, the prevalence of dementia almost doubles every five years and, as elsewhere,^28^^,^^29^ about half of those who have survived to an age of at least 90 years are affected by dementia. According to our estimates, the economic burden of dementia in China will increase substantially over the next few decades and this increase will be driven primarily by population ageing and the increasing prevalence of dementia. Our estimates of the total costs of dementia in China for 2010 and 2030 represent about 7.8% of the estimated US$ 604 billion global cost in 2010 and 10% of the forecasted US$ 1110 billion global cost in 2030.^1^^,^^7^ Our primary estimates of dementia costs are probably underestimates as they ignore the effects, on the costs of care, of home visits by professional careers, the predicted increases in the prevalence of diabetes, hypertension and other risk factors for dementia^30^ and the predicted increases in the use of medical services and devices.

Our analyses indicated that, in China, informal care accounted for more than 80% of the total dementia costs in 2010. This proportion is consistent with the global trend revealed by a systematic review^31^ but higher than the proportion, of about 60%, reported for low- and middle-income countries by the World Health Organization.^32^ In China, the care of an elderly family member is traditionally perceived as an act of filial piety and dementia cases are therefore generally kept out of nursing homes.^15^^,^^33^ In rural areas there is also a lack of facilities and professional carers for dementia cases.^34^ There is no consensus on how to estimate the costs of informal care – with regard to care hours, costs per hour or types of caregiver – or the potential indirect costs, e.g. of lost productivity, incurred by informal caregivers.^7^^,^^13^^,^^20^ As in previous estimates of the global costs of dementia,^7^^,^^13^ we assumed that informal caregivers would be earning the national mean wage if they were not caring for dementia cases. Our estimates of the costs for hospitalization due to dementia indicated that such costs increased more slowly between 2000 and 2010 than between 1990 and 2000. It seems likely that hospitalization costs became more stable in 2003, when the Chinese government launched a programme of health-care reform designed to make medical treatment more accessible.^35^ Our data also revealed a substantial increase in the proportion of the total costs of dementia care attributed to formal care between 2000 and 2010. In countries with rapid economic growth, the responsiveness of the quantity demanded for a service to a change in the income of the people demanding that service, that is, the income elasticity of demand, usually increases the use of formal care. Given the Chinese tradition of home care and the debate over whether formal care can ever meet all of the emotional and psychological needs of dementia cases,^36^^,^^37^ it remains to be seen whether increasing wealth in China will have much effect on the uptake of formal care for dementia cases. However, it seems possible that China’s one-child policy, which was implemented in the late 1970s and only phased out from 2015, will leave too few adults in the 2030s to give sustainable informal care to all of the dementia cases.^38^^,^^39^

### Implications for health policy

In China, public health policy needs to be tailored to address the economic burden posed by dementia. There needs to be greater focus on developing an integrated health and social-care system, including improvements in the efficiency of dementia care and improved health education and financial and social support for dementia cases and their caregivers. Dementia care might be integrated with the national programme for critical illness insurance. Informal caregivers could be taught knowledge and skills relevant to dementia care.^40^ Central or local governments could adopt preferential tax policies or offer other financial incentives to encourage nongovernmental organizations to participate more in dementia-related care services and education.

Policy-makers also need to establish a strategic action plan designed to promote the primary and secondary prevention of dementia. In the past few decades, epidemiological studies have identified several modifiable risk factors for dementia, e.g. cardiovascular disease, that can be targeted for primary prevention.^41^ Current intervention strategies against cardiovascular disease are likely to be effective in delaying dementia onset. The declining incidence of dementia in some high-income countries may be attributable to improvements in the control of other risk factors such as diabetes, hypertension and smoking.^42^^–^^45^ In addition, in settings where effective medical and social-care interventions are available, screening for the early detection of dementia may be cost–effective.^46^^,^^47^ Geriatricians in clinical settings should be alert to the first symptoms and signs of the dementia syndrome. Early interventions for dementia may delay entry into nursing homes and reduce the overall costs of care.^48^

### Strengths and limitations

Our estimates were based on data from multiple sources. The cost data on the use of health resources by dementia cases were from itemized routine hospital records, prevalence data were from a comprehensive systematic review^3^ and United Nations population projections and data from the National Bureau of Statistics of China represented the most authoritative sources for demographic and income data. Our study had several limitations. First, given the considerable variations in economy, cultures, health-care systems, social welfare and traditions across China, cost data from two health facilities in Shandong province are unlikely to be nationally representative. We partly addressed this concern by weighting our estimates and performing multiple sensitivity analyses. The values for income per capita, health expenditure and hospital costs for Shandong province are similar to the mean national values.^49^ Second, the growth rate of medical prices is generally faster than that of the GDP values we used to fill gaps in the data on dementia costs. Given the increasing awareness and use of health services for dementia, it also seems likely that the costs of medical services will increase more rapidly in the coming decades than by 5% annually, that is, by the rate we used in our sensitivity analysis. Third, as people with dementia often suffer from other chronic health conditions,^50^^,^^51^ comorbidities might reduce the accuracy of our estimates of dementia costs. Fourth, data on the proportion of patients with dementia seeking professional care were very limited. Finally, while we used a dynamic model in our projection to account for the ageing population and changes in prevalence of dementia over time, alterations in other factors – e.g. use of medical devices, prevalence of risk factors for dementia and the hiring of professional caregivers – may have reduced the accuracy of our predictions. If, in the future, additional data become available, alternative approaches such as micro-simulation may provide better cost estimates.^52^^,^^53^

In conclusion, demographic and epidemiological transitions in the past two decades have driven substantial growth in the economic costs of dementia in China. This trend is likely to continue over the next two decades. Given the huge economic burden of dementia, policy-makers in China are advised to make dementia a national health priority and to develop a strategic nationwide action plan. Failure to take appropriate action now will allow the economic burden of dementia to grow even further and could, in the long-term, cause dysfunction throughout China’s entire health-care system.

## References

[1] Policy brief for heads of government: the global impact of dementia 2013-2050. London: Alzheimer’s Disease International; 2013. Available from: www.alz.co.uk/research/GlobalImpactDementia2013.pdf [cited 2015 Apr 10].

[2] Zhang Y, Xu Y, Nie H, Lei T, Wu Y, Zhang L, et al. Prevalence of dementia and major dementia subtypes in the Chinese populations: a meta-analysis of dementia prevalence surveys, 1980–2010. J Clin Neurosci. 2012 10;19(10):1333–7. 10.1016/j.jocn.2012.01.029 22682650

[3] Chan KY, Wang W, Wu JJ, Liu L, Theodoratou E, Car J, et al.; Global Health Epidemiology Reference Group (GHERG). Epidemiology of Alzheimer’s disease and other forms of dementia in China, 1990–2010: a systematic review and analysis. Lancet. 2013 6 8;381(9882):2016–23. 10.1016/S0140-6736(13)60221-4 23746902

[4] Wu YT, Lee HY, Norton S, Prina AM, Fleming J, Matthews FE, et al. Period, birth cohort and prevalence of dementia in mainland China, Hong Kong and Taiwan: a meta-analysis. Int J Geriatr Psychiatry. 2014 12;29(12):1212–20. 10.1002/gps.4148 24854229 PMC4552972

[5] Jia J, Wang F, Wei C, Zhou A, Jia X, Li F, et al. The prevalence of dementia in urban and rural areas of China. Alzheimers Dement. 2014 1;10(1):1–9. 23871765 10.1016/j.jalz.2013.01.012

[6] Zhang NJ, Guo M, Zheng X. China: awakening giant developing solutions to population aging. Gerontologist. 2012 10;52(5):589–96. 10.1093/geront/gns105 22936537

[7] Wimo A, Jönsson L, Bond J, Prince M, Winblad B; Alzheimer Disease International. The worldwide economic impact of dementia 2010. Alzheimers Dement. 2013 1;9(1):1–11, e3. 10.1016/j.jalz.2012.11.006 23305821

[8] Sköldunger A, Wimo A, Johnell K. Net costs of dementia in Sweden – an incidence based 10 year simulation study. Int J Geriatr Psychiatry. 2012 11;27(11):1112–7. 10.1002/gps.2828 22298311

[9] Knapp M, Prince M. Dementia UK. London: Alzheimer’s Society; 2007. Available from: www.alzheimers.org.uk/site/scripts/download_info.php?fileID=2323[cited 2015 Mar 12].

[10] Oremus M, Aguilar SC. A systematic review to assess the policy-making relevance of dementia cost-of-illness studies in the US and Canada. Pharmacoeconomics. 2011 2;29(2):141–56. 10.2165/11539450-000000000-00000 21090840

[11] Hurd MD, Martorell P, Delavande A, Mullen KJ, Langa KM. Monetary costs of dementia in the United States. N Engl J Med. 2013 4 4;368(14):1326–34. 10.1056/NEJMsa1204629 23550670 PMC3959992

[12] Olesen J, Gustavsson A, Svensson M, Wittchen HU, Jönsson B; CDBE2010 study group; European Brain Council. The economic cost of brain disorders in Europe. Eur J Neurol. 2012 1;19(1):155–62. 22175760 10.1111/j.1468-1331.2011.03590.x

[13] World Alzheimer Report 2010: the global economic impact of dementia. London: Alzheimer’s Disease International; 2010. Available from: http://www.alz.co.uk/research/files/WorldAlzheimerReport2010.pdf [cited 2015 Apr 21].

[14] The international statistical classification of diseases and related health problem, tenth revision. Geneva: World Health Organization; 1992. Available from: http://www.who.int/classifications/icd/en/ [cited 2015 Apr 21].

[15] Wang G, Cheng Q, Zhang S, Bai L, Zeng J, Cui PJ, et al. Economic impact of dementia in developing countries: an evaluation of Alzheimer-type dementia in Shanghai, China. J Alzheimers Dis. 2008 9;15(1):109–15. 18780971 10.3233/jad-2008-15109

[16] China statistical yearbook 2015. Beijing: National Bureau of Statistics of China; 2015. Available from: http://www.stats.gov.cn/tjsj/ndsj/2015/indexeh.htm [cited 2015 Apr 21].

[17] United Nations Population Information Network [Internet]. New York: United Nations; 2015. Available from: www.un.org/popin [cited 2015 Apr 21].

[18] Chen LL, Zhao GM, Tang JK, Fang H. A study on the economic burden of people with senile dementia in nursing homes. Chin Health Econ. 2009;28(11):19–21. Chinese.

[19] Hu WS, Tang MN, Zheng HB, Ma C, Hu HY. Study on economic burden of senile dementia in community, nursing institution and psychiatric hospital. J Pract Med. 2008;24(10):1821–3. Chinese.

[20] Wang H, Gao TF, Wimo A, Yu X. Caregiver time and cost of home care for Alzheimer’s disease: a clinic-based observational study in Beijing, China. Ageing Int. 2010;35(2):153–65. 10.1007/s12126-010-9056-1

[21] Phillips MR, Zhang J, Shi Q, Song Z, Ding Z, Pang S, et al. Prevalence, treatment, and associated disability of mental disorders in four provinces in China during 2001–05: an epidemiological survey. Lancet. 2009 6 13;373(9680):2041–53. 10.1016/S0140-6736(09)60660-7 19524780

[22] Reisberg B, Ferris SH, de Leon MJ, Crook T. The Global Deterioration Scale for assessment of primary degenerative dementia. Am J Psychiatry. 1982 9;139(9):1136–9. 10.1176/ajp.139.9.1136 7114305

[23] Li H, Huang PR. The disability weights and burden of inpatients with dementia. Chin J Gerontol. 2010;30:243–5. Chinese.

[24] Luengo-Fernandez R, Leal J, Gray A, Sullivan R. Economic burden of cancer across the European Union: a population-based cost analysis. Lancet Oncol. 2013 11;14(12):1165–74. 10.1016/S1470-2045(13)70442-X 24131614

[25] Ruwaard D, Hoogenveen RT, Verkleij H, Kromhout D, Casparie AF, van der Veen EA. Forecasting the number of diabetic patients in the Netherlands in 2005. Am J Public Health. 1993 7;83(7):989–95. 10.2105/AJPH.83.7.989 8328622 PMC1694793

[26] Ang BW. Decomposition analysis for policymaking in energy: which is the preferred method? Energy Policy. 2004;32(9):1131–9. 10.1016/S0301-4215(03)00076-4

[27] Cheng XM. Health economics. 2nd ed. Beijing: People’s Medical Publishing House; 2007. p. 90. Chinese.

[28] Corrada MM, Brookmeyer R, Berlau D, Paganini-Hill A, Kawas CH. Prevalence of dementia after age 90: results from the 90+ study. Neurology. 2008 7 29;71(5):337–43. 10.1212/01.wnl.0000310773.65918.cd 18596243

[29] Lucca U, Tettamanti M, Logroscino G, Tiraboschi P, Landi C, Sacco L, et al. Prevalence of dementia in the oldest old: the Monzino 80-plus population based study. Alzheimers Dement. 2015 3;11(3):258–70.e3. 25150732 10.1016/j.jalz.2014.05.1750

[30] Yang G, Wang Y, Zeng Y, Gao GF, Liang X, Zhou M, et al. Rapid health transition in China, 1990–2010: findings from the Global Burden of Disease Study 2010. Lancet. 2013 6 8;381(9882):1987–2015. 23746901 10.1016/S0140-6736(13)61097-1 PMC7159289

[31] Schaller S, Mauskopf J, Kriza C, Wahlster P, Kolominsky-Rabas PL. The main cost drivers in dementia: a systematic review. Int J Geriatr Psychiatry. 2015 2;30(2):111–29. 10.1002/gps.4198 25320002

[32] Dementia: a public health priority. Geneva: World Health Organization; 2012. Available from: http://www.who.int/mental_health/publications/dementia_report_2012/en/ [cited 2015 Apr 21].

[33] Zhu CW, Scarmeas N, Torgan R, Albert M, Brandt J, Blacker D, et al. Clinical features associated with costs in early AD: baseline data from the Predictors Study. Neurology. 2006 4 11;66(7):1021–8. 10.1212/01.wnl.0000204189.18698.c7 16606913

[34] Wu C, Gao L, Chen S, Dong H. Care services for elderly people with dementia in rural China: a case study. Bull World Health Organ. 2016 3 1;94(3):167–73. 10.2471/BLT.15.160929 26966327 PMC4773933

[35] Wagstaff A, Yip W, Lindelow M, Hsiao WC. China’s health system and its reform: a review of recent studies. Health Econ. 2009 7;18(2) Suppl 2:S7–23. 19551753 10.1002/hec.1518

[36] Bakker C, de Vugt ME, van Vliet D, Verhey FR, Pijnenburg YA, Vernooij-Dassen MJ, et al. The use of formal and informal care in early onset dementia: results from the NeedYD study. Am J Geriatr Psychiatry. 2013 1;21(1):37–45. 23290201 10.1016/j.jagp.2012.10.004

[37] Jiménez-Martín S, Prieto CV. The trade-off between formal and informal care in Spain. Eur J Health Econ. 2012 8;13(4):461–90. 21584815 10.1007/s10198-011-0317-z

[38] Flaherty JH, Liu ML, Ding L, Dong B, Ding Q, Li X, et al. China: the aging giant. J Am Geriatr Soc. 2007 8;55(8):1295–300. 10.1111/j.1532-5415.2007.01273.x 17661972

[39] Glass AP, Gao Y, Luo J. China: facing a long-term care challenge on an unprecedented scale. Glob Public Health. 2013 7;8(6):725–38. 23600434 10.1080/17441692.2013.782060

[40] Jensen M, Agbata IN, Canavan M, McCarthy G. Effectiveness of educational interventions for informal caregivers of individuals with dementia residing in the community: systematic review and meta-analysis of randomised controlled trials. Int J Geriatr Psychiatry. 2015 2;30(2):130–43. 10.1002/gps.4208 25354132

[41] Qiu C, Fratiglioni L. A major role for cardiovascular burden in age-related cognitive decline. Nat Rev Cardiol. 2015 5;12(5):267–77. 10.1038/nrcardio.2014.223 25583619

[42] Qiu C, von Strauss E, Bäckman L, Winblad B, Fratiglioni L. Twenty-year changes in dementia occurrence suggest decreasing incidence in central Stockholm, Sweden. Neurology. 2013 5 14;80(20):1888–94. 10.1212/WNL.0b013e318292a2f9 23596063

[43] Matthews FE, Stephan BC, Robinson L, Jagger C, Barnes LE, Arthur A, et al.; Cognitive Function and Ageing Studies (CFAS) Collaboration. A two decade dementia incidence comparison from the Cognitive Function and Ageing Studies I and II. Nat Commun. 2016;7:11398. 10.1038/ncomms11398 27092707 PMC4838896

[44] Satizabal CL, Beiser AS, Chouraki V, Chêne G, Dufouil C, Seshadri S. Incidence of dementia over three decades in the Framingham Heart Study. N Engl J Med. 2016 2 11;374(6):523–32. 10.1056/NEJMoa1504327 26863354 PMC4943081

[45] Winblad B, Amouyel P, Andrieu S, Ballard C, Brayne C, Brodaty H, et al. Defeating Alzheimer’s disease and other dementias: a priority for European science and society. Lancet Neurol. 2016 4;15(5):455–532. 26987701 10.1016/S1474-4422(16)00062-4

[46] Dixon J, Ferdinand M, D’Amico F, Knapp M. Exploring the cost-effectiveness of a one-off screen for dementia (for people aged 75 years in England and Wales). Int J Geriatr Psychiatry. 2015 5;30(5):446–52. 10.1002/gps.4158 25043227

[47] Brayne C, Fox C, Boustani M. Dementia screening in primary care: is it time? JAMA. 2007 11 28;298(20):2409–11. 18042918 10.1001/jama.298.20.2409

[48] Banerjee S, Wittenberg R. Clinical and cost effectiveness of services for early diagnosis and intervention in dementia. Int J Geriatr Psychiatry. 2009 7;24(7):748–54. 19206079 10.1002/gps.2191

[49] Xu J, Wang J, Wimo A, Qiu C. The economic burden of mental disorders in China, 2005–2013: implications for health policy. BMC Psychiatry. 2016;16(1):137. 10.1186/s12888-016-0839-0 27169936 PMC4864926

[50] Bunn F, Burn AM, Goodman C, Rait G, Norton S, Robinson L, et al. Comorbidity and dementia: a scoping review of the literature. BMC Med. 2014;12:192. 25358236 10.1186/s12916-014-0192-4 PMC4229610

[51] Poblador-Plou B, Calderón-Larrañaga A, Marta-Moreno J, Hancco-Saavedra J, Sicras-Mainar A, Soljak M, et al. Comorbidity of dementia: a cross-sectional study of primary care older patients. BMC Psychiatry. 2014;14(1):84. 10.1186/1471-244X-14-84 24645776 PMC3994526

[52] Brookmeyer R, Evans DA, Hebert L, Langa KM, Heeringa SG, Plassman BL, et al. National estimates of the prevalence of Alzheimer’s disease in the United States. Alzheimers Dement. 2011 1;7(1):61–73. 10.1016/j.jalz.2010.11.007 21255744 PMC3052294

[53] Norton S, Matthews FE, Brayne C. A commentary on studies presenting projections of the future prevalence of dementia. BMC Public Health. 2013;13(1):1. 23280303 10.1186/1471-2458-13-1 PMC3547813

